# Acute Neuromuscular and Hormonal Responses to Power, Strength, and Hypertrophic Protocols and Training Background

**DOI:** 10.3389/fspor.2022.919228

**Published:** 2022-07-14

**Authors:** Johanna Kotikangas, Simon Walker, Sara Toivonen, Heikki Peltonen, Keijo Häkkinen

**Affiliations:** ^1^Biology of Physical Activity, Faculty of Sport and Health Sciences, University of Jyväskylä, Jyväskylä, Finland; ^2^NeuroMuscular Research Center, Jyväskylä, Finland

**Keywords:** resistance training, neuromuscular fatigue, hormones, endocrine system, recovery, training background

## Abstract

This study investigated how two slightly different athlete groups would differ in acute neuromuscular and endocrine responses to specific resistance exercise loadings and recovery compared to untrained participants. Power athletes (PA, *n* = 8), strength athletes (SA, *n* = 8) and non-athletes (NA, *n* = 7) performed power (PL, 7 × 6 × 50% of 1RM), maximal strength (MSL, 7 × 3 × 3RM), and hypertrophic (HL, 5 × 10 × 10RM) loadings in Smith-machine back-squat. Neuromuscular performance, serum testosterone, growth hormone, and cortisol concentrations, and blood lactate (BL) were measured before (Pre), at Mid and after (Post) loading, and after recovery for 24 and 48 h. All loadings led to acute decreases in neuromuscular performance and elevations in hormone concentrations and BL. During PL, a significant group × time interactions occurred in maximal isometric force [*F*_(4, 40)_ = 4.189, *p* = 0.006, $\eta_{\text{p}}^{2}$ = 0.295] indicating a greater decrease in PA compared to SA from Pre to Mid (*p* < 0.05), and in countermovement jump height [*F*_(4, 40)_ = 2.895, *p* = 0.034, $\eta_{\text{p}}^{2}$ = 0.224] indicating a greater decrease in NA compared to SA from Pre to Mid (*p* < 0.05). During HL, growth hormone was higher in Mid and Post in SA compared to NA (*p* < 0.05). No significant interactions were found during recovery. The differences during PL and HL suggest that the training background may enhance acute responses during the present loadings, whereas it seemed to have a limited effect on the recovery.

## Introduction

The variation of resistance exercise variables leads to differing acute responses in the neuromuscular and endocrine systems (Crewther et al., 2006; Corradi et al., 2021). The overall volume (Häkkinen and Pakarinen, 1993; Linnamo et al., 1998; Bartolomei et al., 2017), intensity (Raastad et al., 2000; McCaulley et al., 2009; Bartolomei et al., 2017), time under tension (Wilk et al., 2018; Corradi et al., 2021) and the rest period between sets (Kraemer and Ratamess, 2005) are some variables affecting the magnitude of acute neuromuscular and endocrine responses. Hypertrophic loadings (HL) are typically performed with high volume, moderate intensity, slower movement tempo, and short rest periods between sets and they have resulted in considerable acute decrements in strength and power performance (Walker et al., 2011; Bartolomei et al., 2017) concurrently with large acute increases in serum hormone concentrations and blood lactate (Häkkinen and Pakarinen, 1993; Kraemer and Ratamess, 2005; Linnamo et al., 2005; Kraemer et al., 2017). Maximal strength loadings (MSL) involve lower volume, but the higher intensity and longer rest periods, and significant reductions in maximal muscle activity and voluntary activation along with reductions in force production have been reported during these loadings (Häkkinen, 1993, 1994; McCaulley et al., 2009; Peltonen et al., 2013; Thomas et al., 2018). Small acute decrements or even some increases in neuromuscular performance have been reported during power loadings (PL), which typically are performed with low to moderate volume and intensity, fast movement tempo, and moderate rest periods (McCaulley et al., 2009; Peltonen et al., 2013; Howatson et al., 2016; Thomas et al., 2018). In addition, during PLs, non-significant increases in serum concentration of testosterone, cortisol, and blood lactate have been observed in untrained subjects (Linnamo et al., 2005) and no changes in salivary testosterone or cortisol levels occurred in recreationally weight-trained males (Crewther et al., 2008). Thus, the manipulation of the resistance exercise variables induces differing acute responses, and when resistance exercise is repeated over time these acute responses influence long-term training adaptations (Crewther et al., 2006; Impellizzeri et al., 2019; Corradi et al., 2021).

The training background may also affect the neuromuscular and endocrine responses during resistance exercise and recovery after the exercise (Häkkinen and Myllylä, 1990; Garrandes et al., 2007; Ahtiainen and Häkkinen, 2009; Howatson et al., 2016; Mackey et al., 2018). The previous literature has demonstrated that athletes could elicit greater acute responses to resistance exercise (Häkkinen and Pakarinen, 1993; Ahtiainen et al., 2004; Ahtiainen and Häkkinen, 2009; Crewther et al., 2011). For instance, greater decreases in maximal force and rapid force production have been observed in power and strength athletes compared to habitually active men after fatiguing loading (Häkkinen and Myllylä, 1990). In addition, Ahtiainen and Häkkinen (2009) observed a significant decrease in EMG activity during the concentric phase of action during HL in strength athletes but not non-athletes. During HL, the acute increase in serum testosterone concentration has been reported to be greater in strength athletes compared to non-athletes (Ahtiainen et al., 2004; Tremblay et al., 2004). These differences are probably due to different long-term training adaptations in athletes (Häkkinen and Myllylä, 1990; Brandon et al., 2015), for instance, enhanced tolerance and trainability to specific exercise (Crewther et al., 2006; Brandon et al., 2015), greater neural activation and ability to recruit additional motor units (Häkkinen et al., 1998; Tillin et al., 2010; Brandon et al., 2015) and improved energetic metabolism (Crewther et al., 2006). Thus, these differences may explain the dissimilar neuromuscular and endocrine responses between athletes and non-athletes during different resistance exercises.

Although acute neuromuscular and endocrine responses have been studied broadly during MSLs and HLs and to a limited extent during PLs, still very few studies have compared all of them in the same study. Previous studies have also primarily focused on neuromuscular fatigue and endocrine responses in untrained subjects, and information related to the high-level athletes differing with regard to their training background is sparser (Brandon et al., 2015; Bartolomei et al., 2017; Thomas et al., 2018). For instance, there is no earlier scientific information about possible differences in serum hormone responses between athletes and non-athletes during PL and MSL. The improved understanding of acute responses may help to understand the development of long-term training adaptations caused by the different resistance exercise protocols. In addition, the information provided by the present study may be utilized to individualize and optimize the programming and periodization of resistance exercises in populations with differing training backgrounds (Impellizzeri et al., 2019; Jeffries et al., 2022). It was hypothesized, that athletic groups would elicit greater acute responses in neuromuscular (Häkkinen and Myllylä, 1990; Ahtiainen and Häkkinen, 2009) and endocrine systems (Ahtiainen et al., 2004; Tremblay et al., 2004) during the present loading protocols and recover faster after the loadings compared to NA (Häkkinen and Myllylä, 1990). Thus, the purpose of the present study was to examine to what extent the training background affects the acute neuromuscular and endocrine responses during PL, MSL, and HL and the recovery over 48 h.

## Materials and Methods

### Study Design

The present study included a familiarization session, 3 resistance exercise sessions, and 6 recovery measurement sessions. Approximately 1 week after the familiarization session, PL, MSL, and HL were performed in this order separated by 1 week. All measurements were performed between 12 p.m. and 9 p.m. and the measurement sessions of each participant were performed at the same time of day (±1.5 h). The order of the loadings was not randomized due to challenges in the schedules. The duration of each loading protocol differed quite a lot, and therefore, it would have been difficult to organize the loadings and recovery measurements of various participants to fit in the 1.5-h measurement window.

When the participants reported to the laboratory for resistance exercise loadings, a standardized warm-up routine was performed. All warm-ups began with 4 min of cycling on a stationary bicycle with self-selected resistance and pedal cadence, and 2 sets with 10 repetitions of body-weight squats. Thereafter, 3 warm-up sets were performed at different loads depending on the loading protocol. Before PL, the sets were: 10 repetitions with a barbell, 7–8 repetitions with 30% of 1-RM, and 5–6 repetitions with 40% of 1-RM. Before MSL they were: 5–6 repetitions with 50% of 1-RM, 3–4 repetitions with 70% of 1-RM, and 2–3 repetitions with 80% of 1-RM. Before HL they were: 10 repetitions with 40% of 1-RM, 8–10 repetitions with 50% of 1-RM, and 8–10 repetitions with 60% of 1-RM.

**Figure 1:**
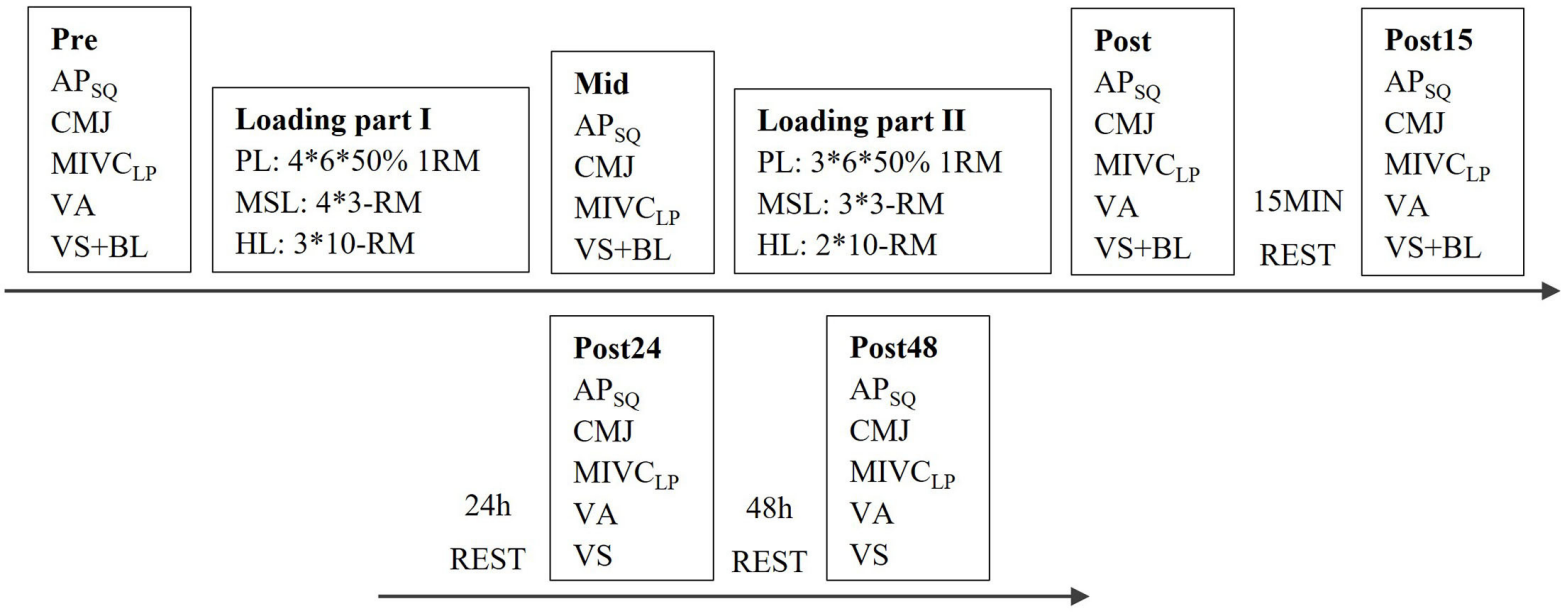
Study design and the measurement procedures during the power (PL), maximal strength (MSL), and hypertrophic (HL) loadings. AP_SQ_, average power during back-squat in Smith machine; CMJ, countermovement jump height; MIVC_LP_, maximal bilateral isometric force in leg press; VA, voluntary activation of quadriceps femoris; VS, venous blood sample; BL, blood lactate concentration.

The neuromuscular, endocrine responses and blood lactate concentrations (BL) were recorded before (Pre), during (Mid, after 3rd or 4th set,), immediately after the loading (Post), and after 15 min (Post 15) of recovery. During a 15 min-rest period, the participants were instructed to relax in a sitting or standing position. The participants also reported to the laboratory after 24 h (Post 24) and 48 h (Post 48) of rest, and the recovery of neuromuscular performance and the concentrations of serum testosterone and cortisol were measured (Figure 1).

The participants were instructed to refrain from any additional training 1 day before and 2 days after the loadings. In addition, the participants were instructed to consume a meal approximately 2–3 h before each measurement session, keep their caffeine intake minimal (at most 1 cup per day), and abstain from the use of any supplements and alcohol the day before measurements and during 3 measurement days of each loading. They were instructed to sleep at least 8 h per night during the measurement weeks, but this was not followed by the researchers. During the measurements, 0.5 dl of water was given to the participants, but they were not allowed to eat anything.

### Subjects

In total 23 healthy men participated in the study. The participants were divided into power athletes (PA, *n* = 8), strength athletes (SA, *n* = 8), and non-athletes (NA, *n* = 7). Mean age was 23.9 y (±2.9) in PA, 24.1 y (±2.7) in SA and 27.6 y (±2.8) in NA. Mean body height and mass were 181.1 cm (±3.6) and 80.5 kg (±8.4), 176.6 cm (±6.3) and 83.2 kg (±13) and 186.6 cm (±7.2) and 93.1 kg (±17.4), respectively. It was required that the athletes had, at least, 3 years of training background from their discipline. PA consisted mainly of track and field athletes, who had systematic training backgrounds primarily in long jump or sprint running and competed at the national level. SA consisted of athletes, who had a training background in the hypertrophic type of resistance training (e.g., bodybuilders and fitness athletes). Some of the participants competed at the national level, but some of the participants did not compete actively or currently. NA did not have a regular resistance training background, but they were mostly recreationally active (participating i.e., a couple of times a week in aerobic training and ball games). Two participants from SA (originally: *n* = 10) and one participant from NA (originally: *n* = 8) did not complete all three loadings and they were excluded from analyses. The reasons for dropouts were the re-occurrence of an earlier musculoskeletal problem (*n* = 2) or personal reasons (*n* = 1). The participants were informed about the study design, and the benefits and risks of the study prior to signing the informed consent document. The study was approved by the Human Sciences Ethics Committee of the University of Jyväskylä and was conducted according to the declaration of Helsinki.

### Loading Protocols

PL consisted of 7 sets with 6 repetitions at the load of 50% of 1-RM and the rest period between sets was 3 min. MSL consisted of 7 sets with 3 repetitions the first set was performed at the load of 90% of 1-RM and the rest period was set for 4 min. HL consisted of 5 sets with 10 repetitions the first set was performed at the load of 70 % of 1-RM and the rest period was set to 1.5 min. These resistance exercise protocols were chosen because they represented typical volumes, intensities, and rest period lengths used in previous resistance exercise studies (Crewther et al., 2006; McCaulley et al., 2009; Walker et al., 2012; Peltonen et al., 2013; Bartolomei et al., 2017; Martorelli et al., 2021). The loading exercise was back-squat in the Smith machine with the lowest knee angle of ~80°. This exercise was chosen because it loads the entire lower extremity and all participants had at least some experience with the back-squat technique. The knee angle was monitored by a custom-built photosensor, which gave an audible signal when the participant reached the appropriate squat depth. During MSL and HL, the load was adjusted after the ending of each set to correspond to actual 3-RM (MSL) and 10-RM (HL). If the participant was able to perform all repetitions without assistance the load was increased or maintained the same and if not, the load was decreased. The participants were instructed to perform all repetitions in every set and if necessary, the spotters helped them with the last repetitions. During PL, the participants were instructed to perform the eccentric phase of back-squat in a controlled manner and the concentric phase at maximal velocity, whereas during MSL and HL they were allowed to select movement tempo independently.

### Familiarization Session and One-Repetition Maximum Test

During the familiarization session, body height and mass were measured and the positions for the surface EMG (sEMG) electrodes of vastus lateralis (VL) and vastus medialis (VM) were determined according to the SENIAM guidelines (Hermens et al., 2000). Thereafter, the devices were set according to the anatomical dimensions of each participant. In the bilateral isometric leg press dynamometer and the unilateral isometric knee extension device, a knee angle of 107° and a hip angle of 110° were determined by a hand-held goniometer and the position was recorded. In the Smith machine, the position of the feet was standardized first and then the knee angle of 80° (i.e., the base of the squat) was measured by a hand-held goniometer (180° = full extension), and the height of the infrared beam of a custom-built photosensor (Faculty of Sport and Health Sciences, University of Jyväskylä, Finland) was adjusted to correspond to the knee angle marking the base position of the back-squat. The one-repetition maximum (1-RM) test of the back-squat in the Smith machine was performed at the end of the familiarization session to determine the loads used during the resistance exercise loadings. Before the 1-RM test, the participants performed a standardized warm-up routine consisting of 4 min biking with a stationary bicycle, 2 sets of 10 repetitions of squats with the barbell, 1 set of 5 to 6 repetitions at the load of 50% of estimated 1-RM, 1 set of 3 to 4 repetitions at the load of 65% of estimated 1-RM and 1 set of 2 repetitions at the load of 80% of estimated 1-RM. The first 1-RM attempt was performed at the load of 85% of the estimated 1-RM and thereafter, the participants performed only one successful repetition with each load (load increased by 2.5 to 10% between attempts) until they could not lift the weight. The estimation of the 1-RM result was requested from each participant before the actual 1-RM test. If the participant of NA was unaware of their estimated 1-RM result, the first warm-up set was performed at a low load (e.g., 40 kg), and the subsequent load increases were estimated by their subjective experience and their performance monitored closely by the researchers.

### Neuromuscular Measures

The following tests to evaluate neuromuscular performance were chosen because they have been utilized systematically in our laboratories and various previous research over several decades (e.g., Häkkinen, 1993; Häkkinen et al., 1998; Ahtiainen and Häkkinen, 2009; Walker et al., 2012; Peltonen et al., 2013).

#### Isometric Testing

Maximal bilateral isometric force (MIVC_LP_) and rapid isometric average force produced during the first 500 ms of the contraction (AF_500_) were assessed in the custom-built bilateral isometric leg press dynamometer (Faculty of Sport and Health Sciences, University of Jyväskylä, Finland). The participants were instructed to push as fast and hard as possible and maintain the maximum effort as long as they were commanded with strong verbal encouragement (approximately 3–5 s). In Pre, Post 24, and Post 48 the participants had three trials with 1-min rest between. In Mid, the participants performed two nearly consecutive trials. In Post and Post 15, three trials were performed with 15–20 s rest between the trials. If the force level of the last trial was 5 percent greater than in the earlier trial, an additional trial was performed at all time points. MIVC_LP_ was defined as the highest value of force (N) recorded during the trials, whereas AF_500_ (N) was calculated in the force-time analysis (Häkkinen et al., 1998).

#### Average Concentric Power

Average concentric power (AP_SQ_) in the maximal power type of back-squat at the load of 50% of 1-RM was performed in the Smith machine. The participants were instructed to lower their bodies toward the ground until they heard the audible signal of the photosensor and then pause their movement approximately for 1 s. After that, the participants were encouraged to push upward as fast as possible. In Pre, Post 24, and Post 48 total of three maximal trials were performed with 1-min rest between trials. In Mid, two consecutive trials were performed so that the participants were allowed to correct their position if they moved during the first trial. Two maximal trials were performed in Post and Post 15 with 15–20 s rest between trials. AP_SQ_ was calculated during the concentric phase of the back-squat by utilizing the data acquired from the infrared sensor to assess the movement of the barbell. The equation used to calculate the average power was P = F × *d*/*t*, where *P* = average power (W), *F* = force (the weight of the external load, N), *d* = the displacement of the barbell (m), *t* = time (s).

#### Countermovement Jump

Countermovement jump height (CMJ) was measured on the force plate (Faculty of Sport and Health Sciences, University of Jyväskylä, Finland). Participants were instructed to lower their bodies rapidly toward the ground and after that change the direction of movement as fast as possible and push off the ground straight upward. The participants were allowed to choose the depth of squat independently, but the hands had to be maintained on the hips throughout the movement. At each test point, 2–3 trials were performed without rest between the trials. The vertical displacement of a participant's center of mass was calculated by using the equation *h* = *I*^2^/(2*gm*^2^), where *h* = vertical displacement of a participant's center of mass, *I* = vertical impulse of force, *g* = acceleration due to gravity, and *m* = mass of the participant.

#### Voluntary Activation

The maximal voluntary activation of the quadriceps femoris muscle (VA) was assessed in a custom-built isometric unilateral knee extension device (Faculty of Sport and Health Sciences, University of Jyväskylä, Finland). Four self-adhesive electrodes with a diameter of 70 mm (Polar Trode, Espoo, Finland) were placed on the proximal and mid regions of the right quadriceps femoris muscle belly. Double 1-ms rectangular pulses with 10-ms inter-stimulus interval were delivered, with increases of 5 mA, by a constant-current stimulator (Model DS7AH, Digitimer Ltd, United Kingdom) during rest until a plateau in twitch force was observed. During the maximal voluntary contraction trials, an additional 30 % of stimulation intensity was added to ensure supramaximal of the stimulus. The participants were asked to generate and maintain maximal force against the ankle strap as long as they were encouraged with the verbal command (approximately 3–4 s). The supra-maximal double-pulse electrical stimulation was delivered three separate times: before the maximal voluntary contraction of knee extensors, during the plateau of maximal voluntary force, and finally to the relaxed muscle (adapted from Merton, 1954). VA was calculated by using the equation $VA=100-D\;\times \;\frac{\left(T_{b}T_{\max}\;\right)}{{\;T}_{DTW}}\;\times \;100$, where *D* = the difference between the torque level just before the double stimulus and maximum torque during the double twitch, *T*_*b*_ = the torque level just before the double twitch, *T*_*max*_ = the maximum torque during the double twitch, *T*_*DTW*_ = the torque signal of the double-twitch response in the relaxed muscle. The correction *D* was used in this equation if the doublet stimulus was not applied exactly during the maximum torque level (Strojnik and Komi, 1998).

#### Surface EMG Measurements

Silver-silver chloride surface electrodes (Ambu BlueSensor N, Copenhagen, Denmark, electrode size L 30 mm × W 22 mm, inter-electrode distance 20 mm, AC impedance 600 Ω) were attached to VL and VM muscles of the right lower limb to record sEMG. A Telemyo 2400R telemetric recording system (Noraxon Inc. Scottsdale, Arizona, USA) with a sampling frequency of 2,000 Hz was used for data collection (500 gain). The EMG signal was transmitted to a receiver box and then fed through an A/D converter (Cambridge Electronic Design Ltd., Cambridge, United Kingdom) to a computer where Signal 4.14 software (Cambridge Electronic Design Ltd., Cambridge, United Kingdom) was used for data recording and further analysis offline. The sEMG data were band-pass filtered (20–350 Hz). Maximum sEMG amplitudes of VL and VM were obtained from the root mean square over the 500–1,500 ms time period during the bilateral isometric leg press (sEMG_LP_) and the concentric phase of the back-squat with the load of 50% of 1-RM (sEMG_SQ_). Thereafter, VL and VM amplitudes were averaged (VL+VM/2) to represent the maximal sEMG of the knee extensors.

### Blood Samples

The samples were collected using sterile needles into serum tubes (Vacuette, Greiner Bio-One International GmbH, Austria) and they were kept for 30 min at room temperature. Thereafter, the samples were centrifuged at 2.2 G RCF (Megafuge 2 R, Heraeus, Germany) for 10 min. Then serum was removed and stored at −20°C until analysis. Testosterone (TES), cortisol (COR), and growth hormone (GH) were analyzed by using chemiluminescence immunometric techniques (Immulite 2000, Siemens, Llanberis, United Kingdom). The sensitivities for serum hormones were TES 0.5 nmol/l, COR 5.5 nmol/l and GH 0.01 μg/l with intra-assay coefficient of variation of TES = 8.2%, COR = 7.9%, and GH = 5.8%.

### Blood Lactate

Lactate concentration was determined by taking capillary blood samples from the fingertip while the participant was seated. The sample was collected into a 20 μL capillary tube, which was placed in a 1 mL hemolyzing solution. All four blood lactate samples were analyzed immediately upon completion of each loading by using a Biosen lactate analyzer (S_line Lab+, EKF Diagnostic, Magdeburg, Germany) according to the manufacturer's instructions.

### Statistical Analyses

The results were analyzed using IBM SPSS Statistics-software version 26 (SPSS, Inc., Chicago, Illinois, USA). The results are presented as group mean values, standard deviations (SD), and percentage changes (%). Before the analyses, the normal distribution of the data was checked using the Shapiro-Wilk test. One-way ANOVA was used to evaluate the expected differences between groups in 1-RM and the baseline values measured in PL, with *post-hoc* comparisons tested by Bonferroni's significance test. Differences in acute responses from Pre to Post were examined by using two-way (group [3] × time [3]) repeated measures ANOVA, and in recovery responses from Post to Post 48 by separate two-way (group [3] × time [4]) repeated measures ANOVA. The *post-hoc* comparison of means over the 3 (Pre to Post) and 4 (Post to Post 48) time points and pairwise comparison between groups in different time points were provided by Bonferroni's significance test. Finally, contrast analyses were used to examine within-group differences between all six-time points (from Pre to Post 48), from Pre to Post, and from Post to Post 48. GH data was not normally distributed, so the between-group differences were analyzed by the Kruskal Wallis test and the differences between time points by Friedman and Wilcoxon tests. The level of significance for all tests was set at *p* ≤ 0.05.

## Results

Maximal force levels of PA and SA were significantly higher compared to NA in the back-squat (*p* = 0.038 and *p* < 0.001, respectively) and in the isometric leg press measured during Pre measurement of PL (*p* = 0.004 and *p* < 0.001, respectively; Table 1). The only significant difference between PA and SA was the ratio between back-squat 1-RM and body mass (*p* = 0.023) so SA was relatively stronger compared to PA.

**Table 1:** Group mean values (±SD) of 1RM in back-squat, 1-RM relative to body mass, and all test procedures measured during the Pre-measurements.

**Variable**	**PA**	**SA**	**NA**
1RM in back squat (kg)	144.6 (±24.2)^*^	176.8 (±31.0)^***^	106.4 (±19.8)
1RM/body mass	1.78 (±0.19)^***^/^∧^	2.13 (±0.23)^***^	1.20 (±0.29)
MIVC_LP_ (N)	4,463 (±708)^**^	4,831 (±682)^***^	3,199 (±514)
AF_500_ (N)	3,125 (±534)^***^	2,689 (±729)^**^	1,739 (±550)
AP_SQ_ (W)	700.6 (±120.8)^***^	776.2 (±120.1)^***^	445.5 (±87.9)
CMJ (cm)	45.9 (±5.2)^***^	42.0 (±3.6)^***^	29.5 (±6.2)

*PA, power athletes; SA, strength athletes; NA, non-athletes. MIVC_LP_, maximal bilateral isometric force in leg press; AF_500_, average force during first 500 ms of isometric contraction in leg press; AP_SQ_, average power during maximal power type of back-squat in Smith machine; CMJ, countermovement jump height*.

* *p < 0.05*,

** *p < 0.01, and*

*** *p < 0.001 refers to significant differences compared to NA and*

∧ *p < 0.05 compared to SA*.

### Maximal Bilateral Isometric Force and SEMG Amplitude in Leg Press

**Figure 2:**
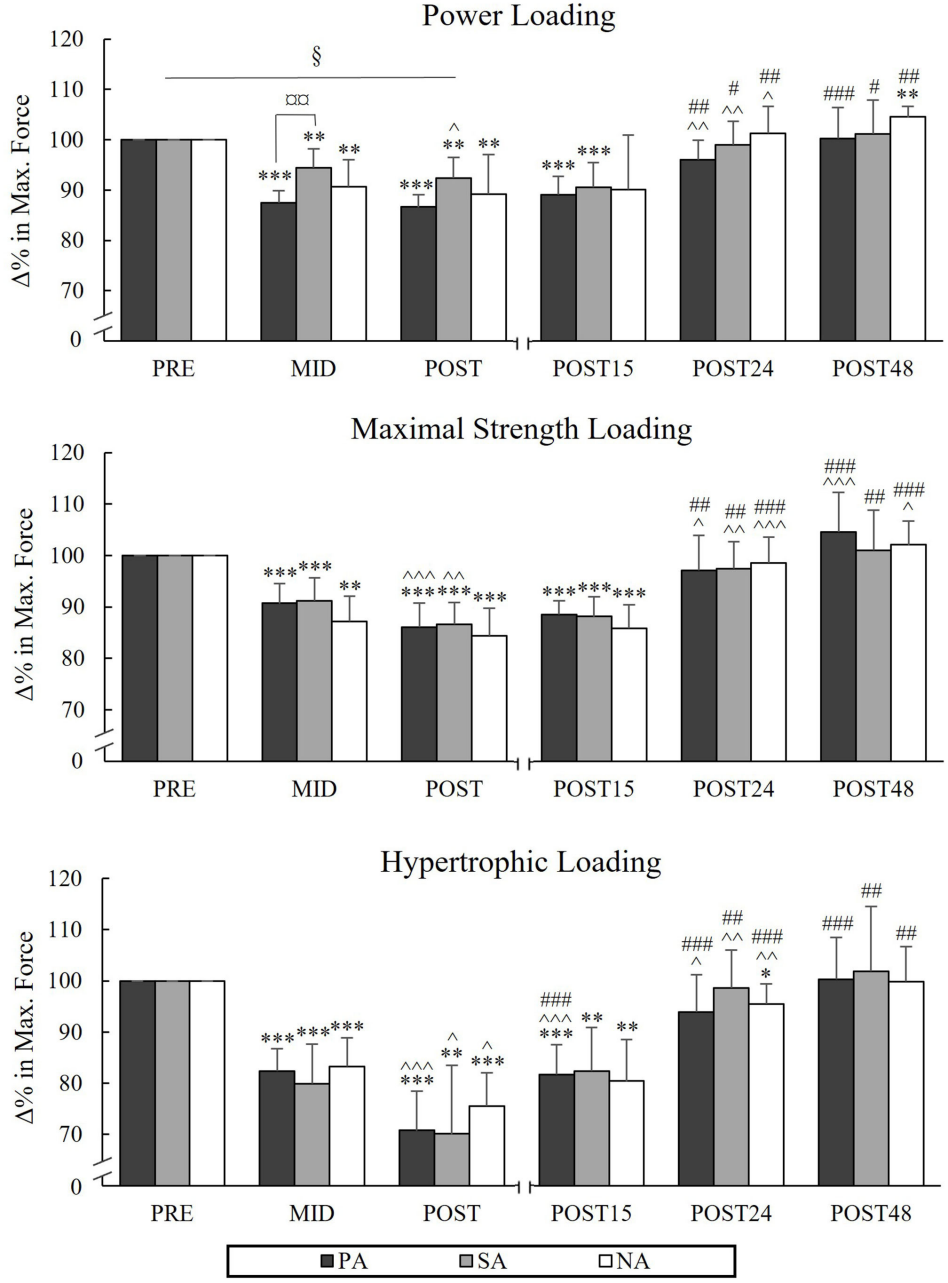
Relative changes in maximal bilateral isometric force during three resistance exercise loadings in power (PA), strength (SA), and non-athletes (NA). *p < 0.05, **p < 0.01, ***p < 0.001 refer to within-group significances compared to Pre. ^∧^*p* < 0.05, ^∧∧^*p* < 0.01, ^∧∧∧^*p* < 0.001 refer to within-group significances compared to the previous time point. #*p* < 0.05, ##*p* < 0.01, ###*p* < 0.001 refer to within-group significances compared to Post during recovery. §*p* < 0.05 refers to significant group × time point interaction from Pre to Post. ¤¤*p* < 0.01 refers to a significant group difference between PA and SA.

**Figure 3:**
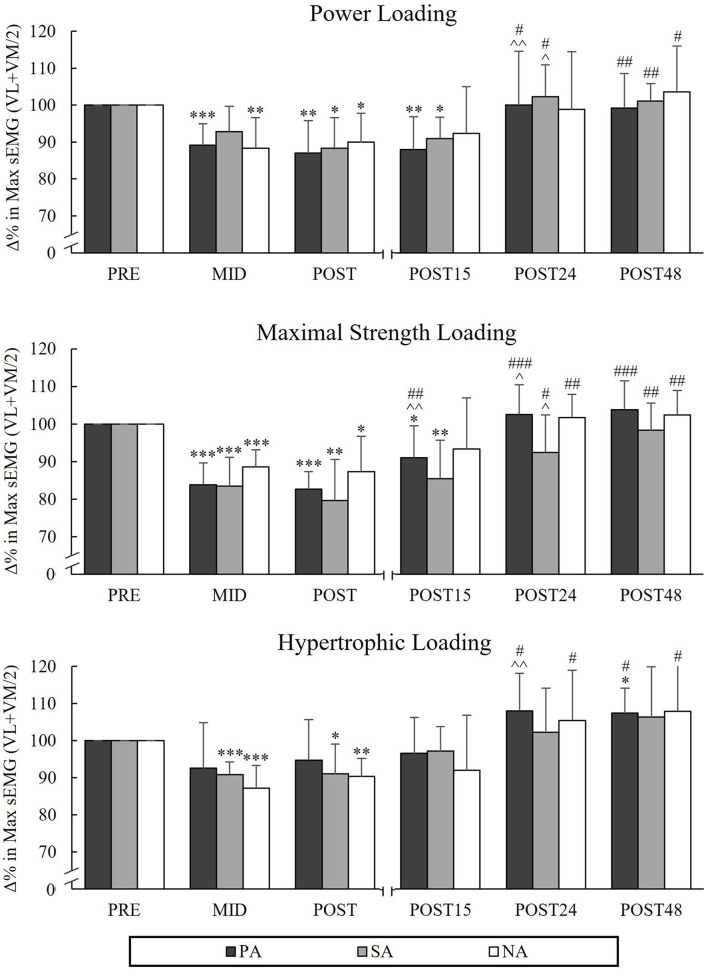
Relative changes in maximal averaged sEMG values of VL and VM muscles in isometric leg press during three resistance exercise loadings in power (PA), strength (SA), and non-athletes (NA). *p < 0.05, **p < 0.01, ***p < 0.001 refer to within-group significances compared to Pre. ^∧^*p* < 0.05, ^∧∧^*p* < 0.01 refer to within-group significances compared to the previous time point. #*p* < 0.05, ##*p* < 0.01, ###*p* < 0.001 refer to within-group significances compared to Post during recovery.

During PL, there was a significant group × time interaction in MIVC_LP_ [*F*_(4, 40)_ = 2.894, *p* = 0.034, $\eta_{\text{p}}^{2}$ = 0.224] indicating that the mean value of Pre to Post time points was significantly lower in PA compared to SA (*p* = 0.017). Pairwise comparison between groups in different time points indicated that MIVC_LP_ at the Mid time point was significantly lower in PA compared to SA (*p* = 0.006; Figure 2). After PL, MIVC_LP_ in SA and NA recovered after 24 h of rest, but in PA it was still decreased (−4%, *p* = 0.022). The greatest relative changes in MIVC_LP_ from Pre to Post were observed during HL (PA −29.2%, *p* < 0.001, *n* = 7, SA −29.9%, *p* = 0.002 and NA −24.5%, *p* < 0.001). After HL, both athlete groups were fully recovered after 24 h of rest, whereas in NA MIVC_LP_ was still declined (−4.5%, *p* = 0.025). Significant decreases in sEMG_LP_ during HL occurred in SA and NA Pre to Post (−9%, *p* = 0.026, *n* = 7, and −9.7%, *p* = 0.002 respectively), but not in PA (−5.3%, *p* = 0.25). The greatest decreases in maximal sEMG_LP_ occurred during MSL from Pre to Post (PA −17.4 %, *p* < 0.001, SA −20.3%, *p* = 0.003, *n* = 7, NA −12.6 %, *p* = 0.012; Figure 3).

### Average Rapid Force During 500 ms

**Figure 4:**
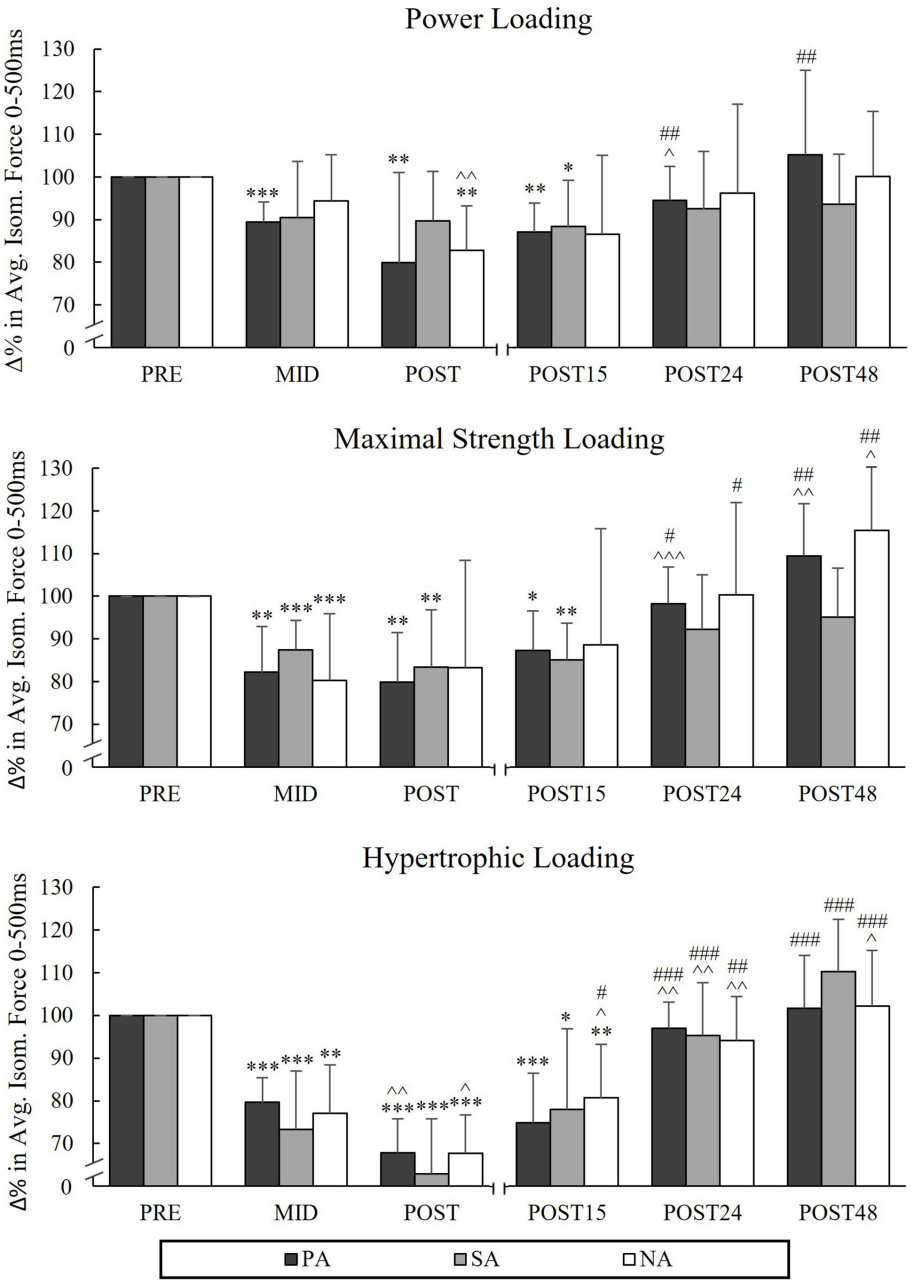
Relative changes in average force in 0–500 ms of maximal isometric leg press during three resistance exercise loadings in power (PA), strength (SA), and non-athletes (NA). *p < 0.05, **p < 0.01, ***p < 0.001 refer to within-group significances compared to Pre. ^∧^*p* < 0.05, ^∧∧^p < 0.01, ^∧∧∧^*p* < 0.001 refer to within-group significances compared to the previous time point. #*p* < 0.05, ##*p* < 0.01, ###*p* < 0.001 refer to within-group significances compared to Post during recovery.

During PL, significant decreases in AF_500_ occurred from Pre to Post in PA (−12.9%, *p* = 0.003) and NA (−17.2%, *p* = 0.029, *n* = 6; Figure 4) and during MSL in the athlete groups (PA −20.1%, *p* = 0.006, SA −16.6%, *p* = 0.004). In HL, significant decreases were observed in AF_500_ in all groups from Pre to Post (−32.1%, *p* < 0.001, *n* = 7 in PA, −37.1%, *p* < 0.001, *n* = 7 in SA and −32.3%, *p* < 0.001 in NA).

### Average Power and SEMG Amplitude During Back-Squat

**Figure 5:**
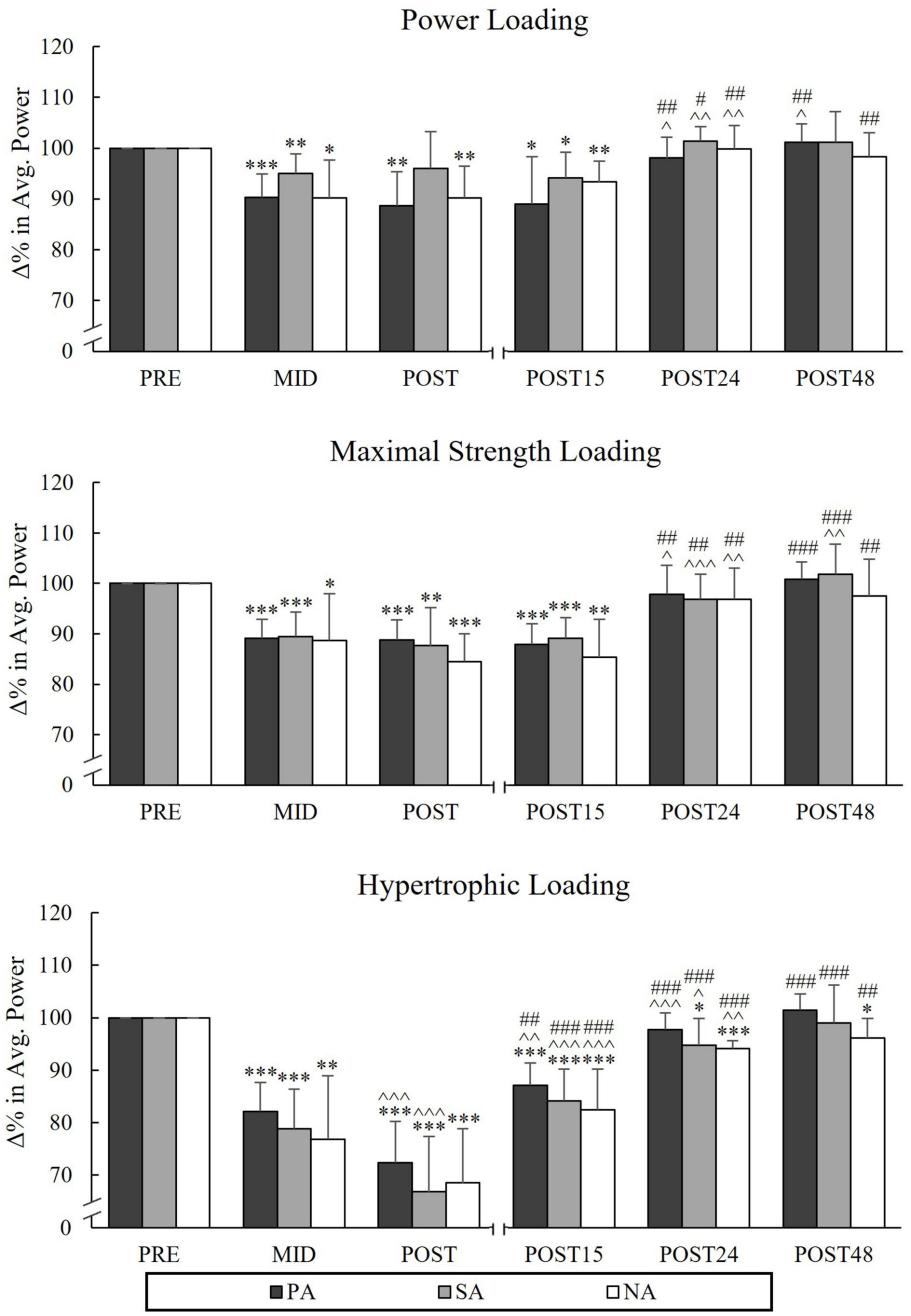
Relative changes in average power during the concentric phase of maximal power back-squat in Smith machine during three resistance exercise loadings in power (PA), strength (SA), and non-athletes (NA). *p < 0.05, **p < 0.01, ***p < 0.001 refer to within-group significances compared to Pre. ^∧^*p* < 0.05, ^∧∧^*p* < 0.01, ^∧∧∧^*p* < 0.001 refer to within-group significances compared to the previous time point. ^#^*p* < 0.05, ^*##*^*p* < 0.01, ^*###*^*p* < 0.001 refer to within-group significances compared to Post during recovery.

**Figure 6:**
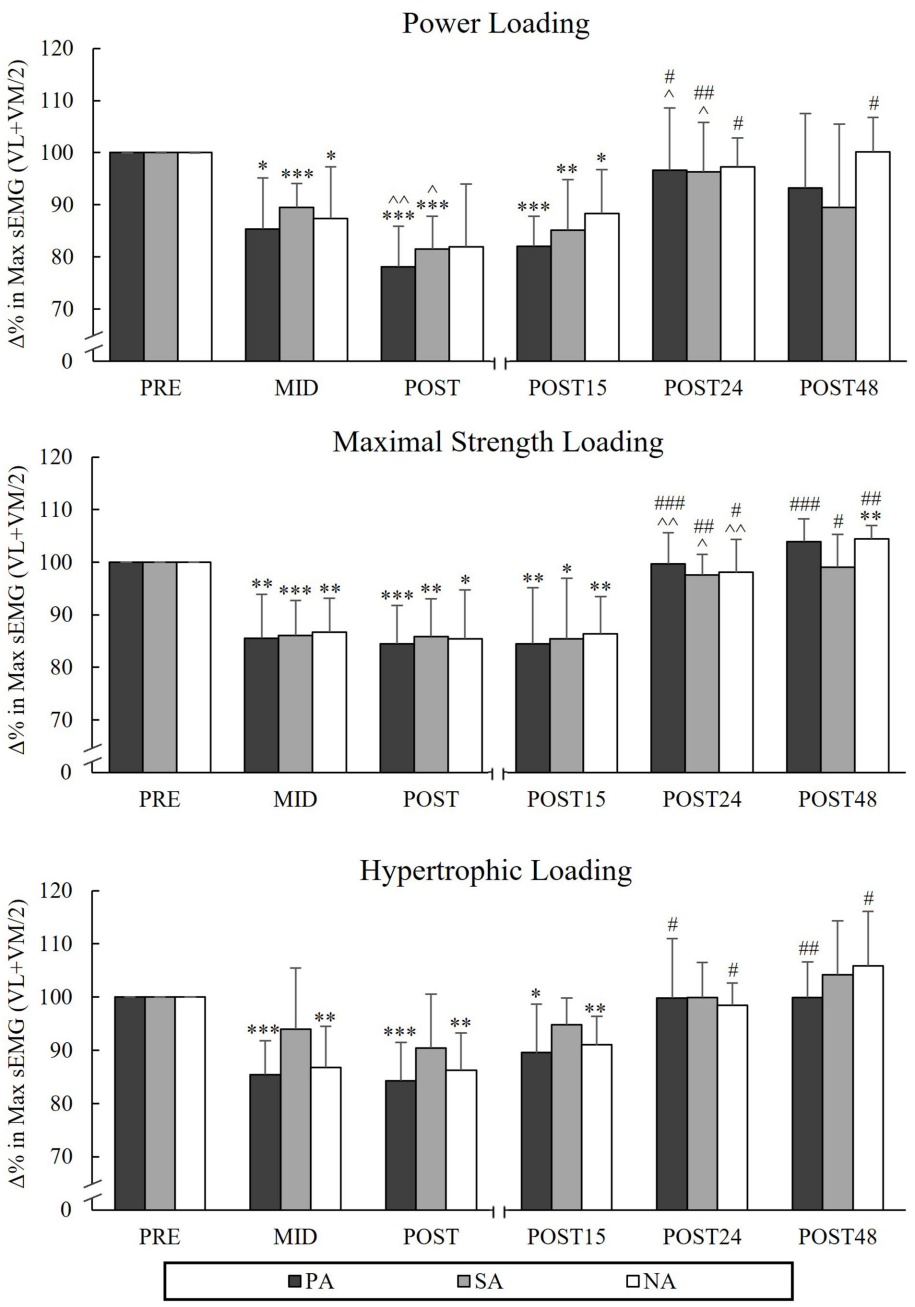
Relative changes in maximal averaged sEMG amplitudes of VL and VM muscles during the concentric phase of maximal power back-squat during three resistance exercise loadings in power (PA), strength (SA), and non-athletes (NA). *p < 0.05, **p < 0.01, ***p < 0.001 refer to within-group significances compared to Pre. ^∧^*p* < 0.05, ^∧∧^*p* < 0.01 refer to within-group significances compared to the previous time point. ^#^*p* < 0.05, ^*##*^*p* < 0.01, ^*###*^*p* < 0.001 refer to within-group significances compared to Post during recovery.

During PL, there were significant reductions from Pre to Post in AP_SQ_ in PA (−11.4%, *p* = 0.002, *n* = 7) and NA (−9.8%, *p* = 0.006), but not in SA (−3.9%, *p* = 0.16; Figure 5). The greatest decreases in AP_SQ_ were observed during HL in all groups from Pre to Post (PA −27.6%, *p* < 0.001, *n* = 7, SA −33.2%, *p* < 0.001 and NA −31.5%, *p* < 0.001) and it continued to decrease from Mid to Post in PA and SA (−9.7%, *p* < 0.001 and −12%, *p* = 0.002, respectively). During HL, significant reductions in sEMG_SQ_ from Pre to Post were observed in PA (−15.7%, *p* = 0.001, *n* = 7) and NA (−13.8%, *p* = 0.002), but not in SA (-9.5%, *p* = 0.068, *n* = 6; Figure 6). After PL and MSL, AP_SQ_ recovered to the baseline after 24 h of rest in all the groups. Only after HL all groups had increase in AP_SQ_ from Post to Post 15 (in PA +15%, *p* = 0.001, *n* = 7, SA +17.3%, *p* = 0.006 and NA +14%, *p* = 0.002). After 24 h of rest, AP_SQ_ remained lower in SA (−5.2%, *p* = 0.038) and NA (−5.8%, *p* < 0.001) and remained lower in NA even after 48 h of rest (−3.9%, *p* = 0.035) compared to Pre.

### Voluntary Activation

**Figure 7:**
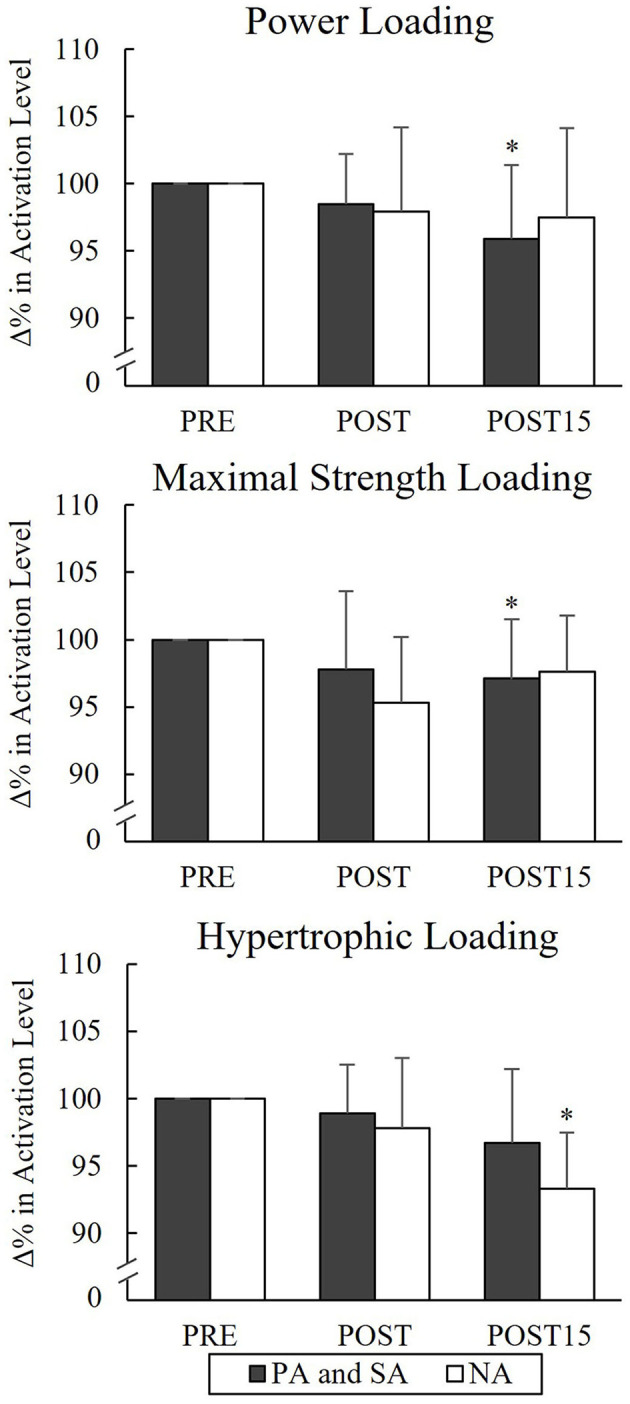
Relative changes in maximal voluntary activation of quadriceps femoris muscle in the combined group of power (PA), strength (SA) athletes, and non-athletes (NA). *p < 0.05 refers to within-group significances compared to Pre.

None of the groups showed significant changes in VA from Pre to Post or Post 15 during PL or MSL, but significant decreases in VA occurred from Pre to Post 15 in the combined athlete group (*n* = 12) during PL (−4%, *p* = 0.024) and MSL (−2.9%, *p* = 0.043; Figure 7). During HL, a significant difference was found from Pre to Post 15 in NA (−6.7%, *p* = 0.012).

### Countermovement Jump

**Figure 8:**
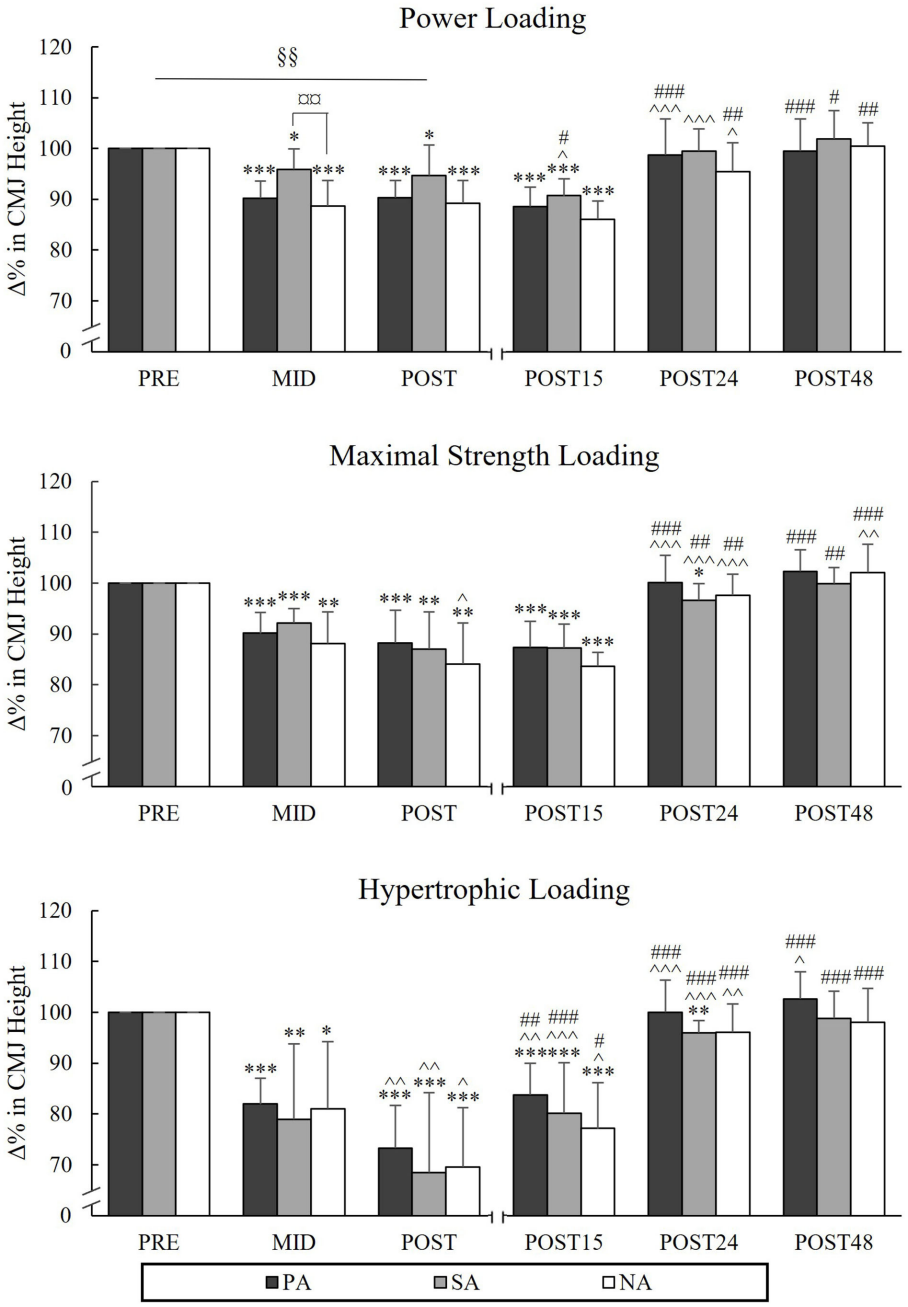
Relative changes in CMJ height during three resistance exercise loadings in power (PA), strength (SA), and non-athletes (NA). *p < 0.05, **p < 0.01, ***p < 0.001 refer to within-group significances compared to Pre. ^∧^*p* < 0.05, ^∧∧^*p* < 0.01, ^∧∧∧^*p* < 0.001 refer to within-group significances compared to previous time point. ^#^*p* < 0.05, ^*##*^*p* < 0.01, ^*###*^*p* < 0.001 refer to within-group significances compared to Post during recovery. §§*p* < 0.01 refers to significant group × time point interaction from Pre to Post. ¤¤*p* < 0.01 refers to a significant group difference between NA and SA.

During PL, there was a significant group × time interaction in CMJ [*F*_(4, 40)_ = 2.805, *p* = 0.038, $\eta_{\text{p}}^{2}$ = 0.219], indicating that the mean value of Pre to Post points was significantly lower in NA compared to SA (*p* = 0.009). Pairwise comparison between groups in different time points indicated that CMJ at the Mid time point was significantly lower in NA compared to SA (*p* = 0.009; Figure 8). CMJ was recovered after 24 h of rest in all groups after PL. The greatest reductions from Pre to Post were observed during HL (PA −26.7%, *p* < 0.001, *n* = 7, SA −31.5%, *p* = 0.001 and NA −30.5% *p* < 0.001) and CMJ continued to decline from Mid to Post in all groups (PA −8.6%, *p* = 0.005, *n* = 7, SA −10.4%, *p* = 0.003 and NA −11.5%, *p* = 0.048). After MSL and HL, PA and NA recovered after 24 h of rest, but the performance level of SA remained lowered at this time point (−3.4%, *p* = 0.021 after MSL and −4.1%, *p* = 0.002 after HL).

### Serum Hormone Concentrations

**Figure 9:**
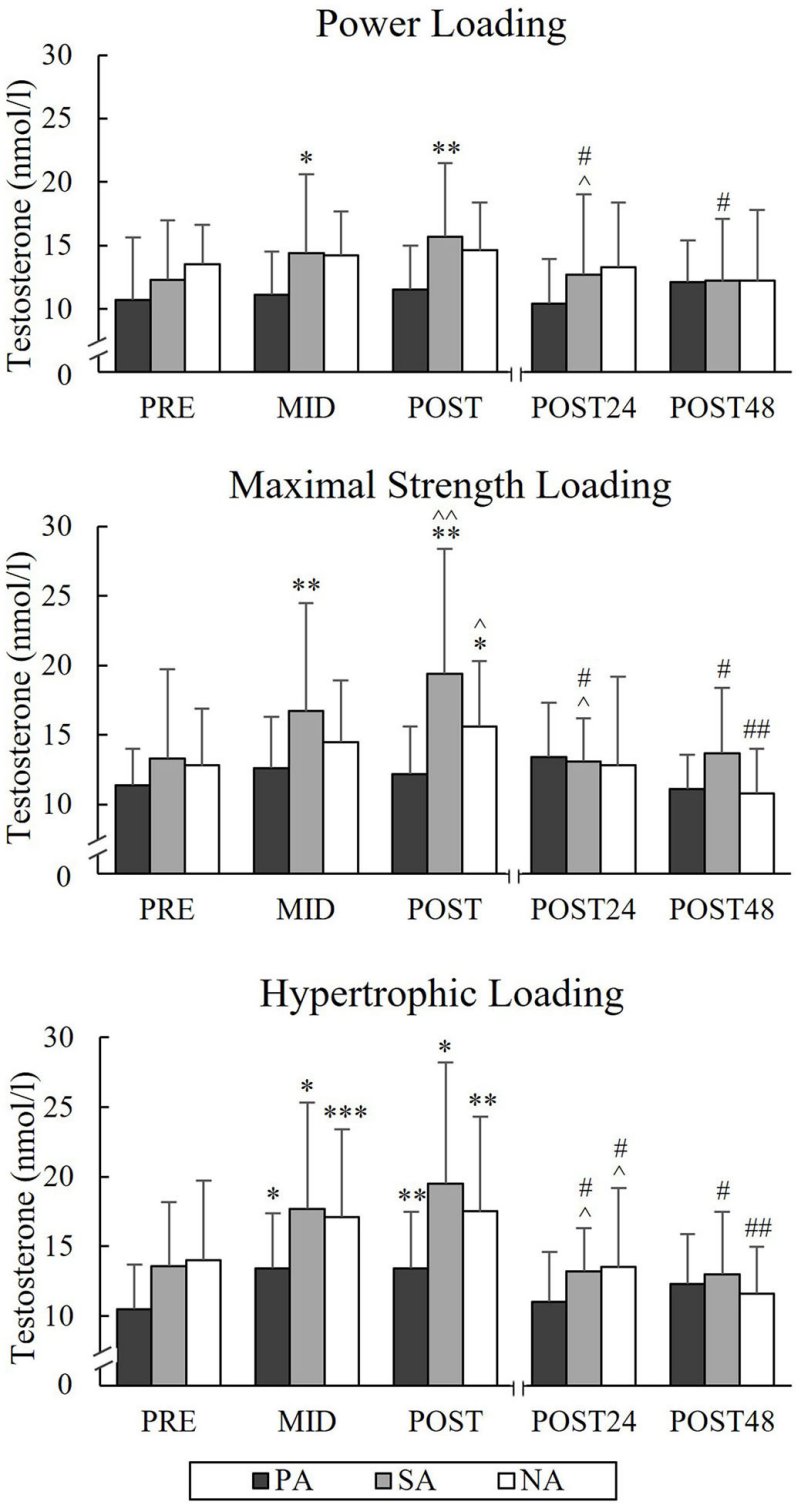
Serum testosterone concentrations of Pre, Mid, Post, Post24, and Post48 during three resistance exercise loadings in power (PA), strength (SA), and non-athletes (NA). *p < 0.05, **p < 0.01, ***p < 0.001 refer to within-group significances compared to Pre. ^∧^*p* < 0.05, ^∧∧^*p* < 0.01 refer to within-group significances compared to the previous time point. ^#^*p* < 0.05, ^*##*^*p* < 0.01 refer to within-group significances compared to Post during recovery.

**Figure 10:**
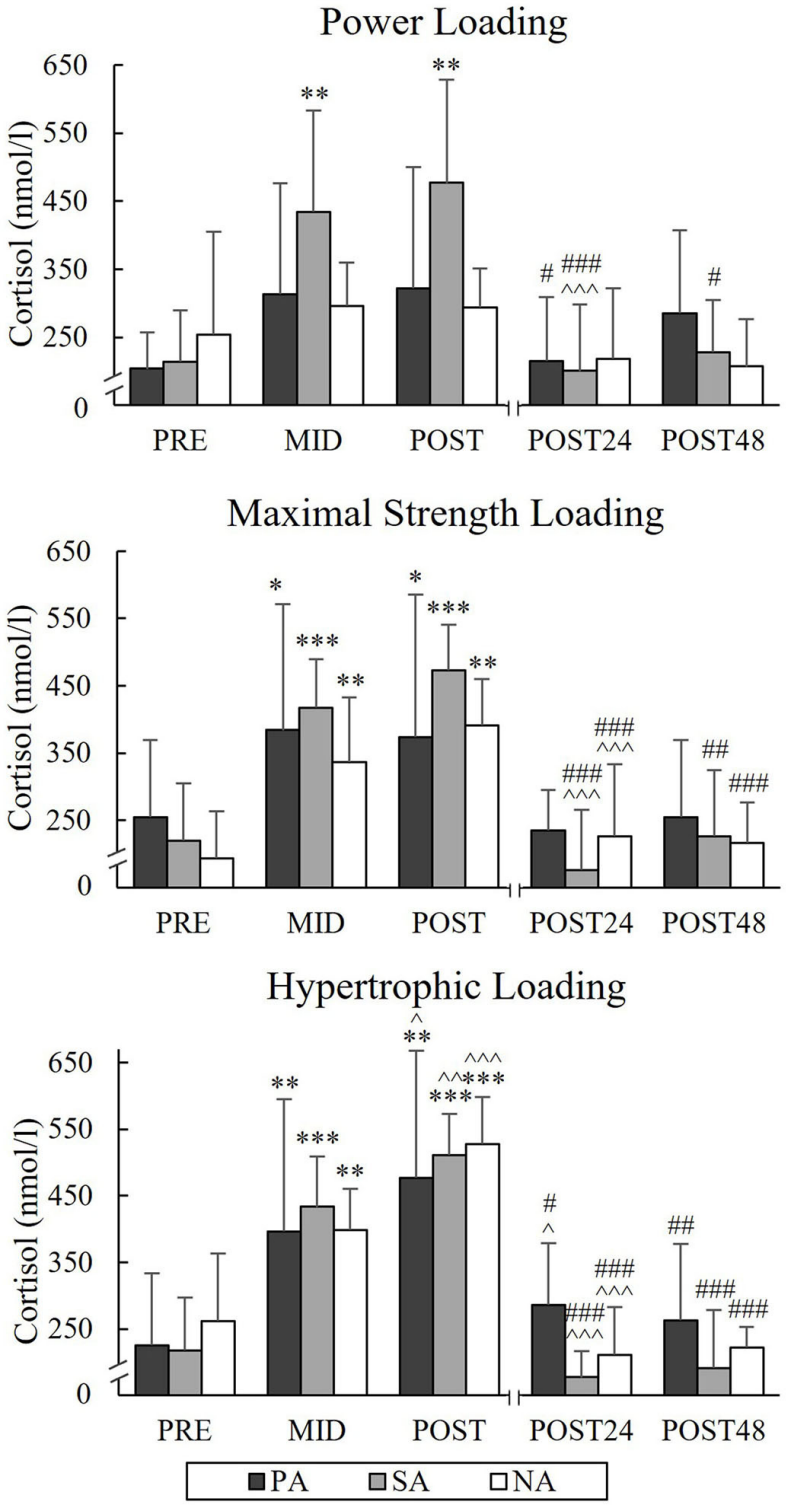
Serum cortisol concentrations of Pre, Mid, Post, Post24, and Post48 during three resistance exercise loadings in power (PA), strength (SA), and non-athletes (NA). *p < 0.05, **p < 0.01, ***p < 0.001 refer to within-group significances compared to Pre. ^∧^*p* < 0.05, ^∧∧^*p* < 0.01, ^∧∧∧^*p* < 0.001 refer to within-group significances compared to the previous time point. ^#^*p* < 0.05, ^*##*^*p* < 0.01, ^*###*^*p* < 0.001 refer to within-group significances compared to Post during recovery.

During PL, TES increased from a Pre-value of 12.3 nmol/l to Post-value of 15.7 nmol/l (*p* = 0.005) and COR from Pre-value of 214.6 nmol/l to Post-value of 476.8 nmol/l (*p* = 0.004) in SA (Figures 9, 10). Significant increases in TES during MSL took place from Pre to Post in SA (from 13.3 to 19.4 nmol/l, *p* = 0.002) and NA (from 12.8 to 15.6 nmol/l, *p* = 0.035). Significant increases in COR were observed in all three groups during MSL from Pre to Post (in PA from 255 to 373.8 nmol/l, *p* = 0.037, in SA from 219.3 to 472.9 nmol/l, *p* = 0.002, and in NA from 193.1 to 391.3 nmol/l, *p* = 0.002). During HL, TES increased significantly from Pre to Post in all groups and Post-values were 13.4 nmol/l in PA (*p* = 0.007, *n* = 7), 19.5 nmol/l in SA (*p* = 0.014) and 17.5 nmol/l in NA (*p* = 0.007). Similarly, COR increased significantly from Pre to Post and Post-values were 477.1 nmol/l in PA (*p* = 0.002, *n* = 7), 511.4 nmol/l in SA (*p* < 0.001) and 527.4 nmol/l in NA (*p* < 0.001). COR still increased from Mid to Post in all three groups. After all loadings, both TES and COR returned to the baseline after 24 h of rest.

**Figure 11:**
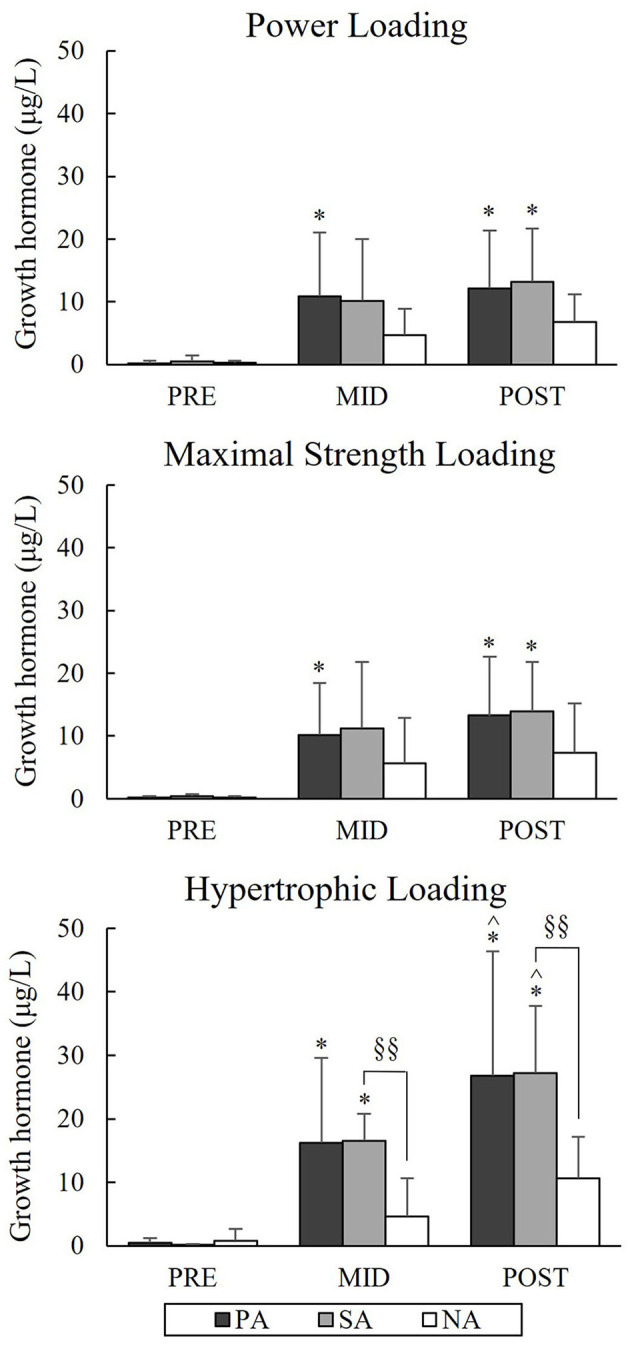
Serum growth hormone concentrations of Pre, Mid, and Post during three resistance exercise loadings in power (PA), strength (SA), and non-athletes (NA). *p < 0.05 refer to within-group significances compared to Pre. ^∧^*p* < 0.05 refer to within-group significances compared to Mid. §§*p* < 0.01 refer to between-group significances.

Serum GH increased significantly in PA and SA from Pre to Post during PL and MSL (Figure 11). In PA this increase was from 0.2 to 12.1 μg/L (*p* = 0.012) during PL and 0.09 to 13.26 μg/L (*p* = 0.012) during MSL. In SA increases were from 0.46 to 13.21 μg/L (*p* = 0.012) and 0.2 to 13.92 μg/L (*p* = 0.012), respectively. During HL, the Post-values of GH were 26.82 μg/L (*p* = 0.012) in PA and 27.23 μg/L (*p* = 0.012) in SA. Group differences between SA and NA were found at Mid (*p* = 0.006) and Post (*p* = 0.003) time points. The relative increases in GH during HL correlated significantly with the relative increases in BL (*r* = 0.63, *p* = 0.001) in the total group of subjects, and also in the separate group of SA (*r* = 0.86, *p* = 0.007).

### Blood Lactate Concentrations

The greatest increases in BL were observed during HL and the difference between Pre and Post was significant in all groups (*p* < 0.001 in all groups; Table 2). Also, significant increases from Pre to Post were observed in all groups after MSL and PL, but they were significantly smaller compared to HL.

**Table 2:** Blood lactate concentrations (mmol/l) (group mean ± SD) during three resistance exercise loadings in power (PA), strength (SA), and non-athletes (NA).

	**Group**	**PRE**	**MID**	**POST**	**POST15**
PL	PA	1.58 (±0.63)	4.31 (±1.63)^***^	3.35 (±1.27)^***^/^∧^/^###^	2.23 (±0.77)
	SA	1.78 (±1.22)	5.07 (±2.82)^*^	4.74 (±2.45)^*^/^∧∧∧^/^###^	2.70 (±1.30)
	NA	2.16 (±0.71)	3.84 (±1.65)^*^	3.86 (±1.63)^*^/^∧∧∧^/^###^	2.49 (±0.86)
MSL	PA	1.59 (±0.67)	5.43 (±2.47)^**^	5.06 (±2.09)^**^/^###^	2.78 (±0.99)
	SA	1.90 (±0.84)	6.39 (±2.64)^**^	7.46 (±2.71)^***^/^###^	3.18 (±1.19)
	NA	1.94 (±0.87)	4.57 (±2.35)^**^	5.60 (±2.10)^***^/^###^	2.36 (±0.86)
HL	PA	1.82 (±1.14)	11.12 (±2.16)^***^	13.76 (±1.86)^***^	5.98 (±2.12)^**^
	SA	1.66 (±0.51)	11.84 (±3.86)^***^	14.37 (±3.27)^***^	6.96 (±3.24)^**^
	NA	2.39 (±1.36)	11.49 (±3.91)^***^	13.68 (±4.06)^***^	7.09 (±3.10)^**^

* *p < 0.05*,

** *p < 0.01, and*

*** *p < 0.001 refer to significant differences compared to Pre*.

∧ *p < 0.05, and*

∧∧∧ *p < 0.001 compared to MSL Post*.

### *p < 0.001 compared to HL Post*.

## Discussion

The present results showed that our three different resistance exercise loadings led to acute specific decrements in neuromuscular performance in all three groups. There were also significant acute elevations in serum hormone concentrations during all resistance exercise loadings in SA, and during MSL and HL in PA and NA. The greatest decreases in neuromuscular performance and greatest elevations in serum hormone concentrations were observed during HL, which agrees well with previous studies (Häkkinen and Pakarinen, 1993; Kraemer and Ratamess, 2005; Linnamo et al., 2005; McCaulley et al., 2009; Bartolomei et al., 2017; Martorelli et al., 2021) and the acute responses after PL and MSL were smaller as was expected (Häkkinen and Pakarinen, 1993; Smilios et al., 2003; McCaulley et al., 2009; Walker et al., 2011; Peltonen et al., 2013). In addition, there were significant acute elevations in TES, COR, and GH after the present MSL, even though the findings regarding acute hormonal responses after MSLs have been somewhat inconsistent (Häkkinen and Pakarinen, 1993; Smilios et al., 2003; Crewther et al., 2008; McCaulley et al., 2009). Significant group × time point interactions were found during PL from Pre to Post so that the decrease in MIVC_LP_ was greater in PA compared to SA and the decrease in CMJ was greater in NA compared to SA. In addition, there were significantly greater elevations in serum GH in SA compared to NA from Pre to Mid and Pre to Post during HL. No significant differences between the groups were observed during the recoveries of any loading protocol.

### Acute Responses During the Loadings

We observed a greater decrease in MIVC_LP_ in PA compared to SA during PL, and also the reductions in AP_SQ_ and CMJ in SA remained minimal. An earlier study by Häkkinen and Myllylä (1990) demonstrated that power athletes can produce force more explosively than strength-trained athletes and Mackey et al. (2018) found, that the early isometric rate of torque development was higher in explosive resistance-trained than traditional resistance-trained men. Thus, our present findings could indicate that PA may have been able to exhaust their neuromuscular system more due to their ability to produce repetitions more explosively and their greater experience from power types of resistance exercise, and reached greater neuromuscular fatigue during PL. In addition, long-term resistance training may cause a transition from type IIx to type IIA muscle fibers (Kraemer et al., 1996; Mackey et al., 2018) and some earlier evidence suggests that a longer time under loading with low loads (<60% of 1-RM) elicits a greater stimulus to type I fibers (Grgic et al., 2018) and these adaptations improve the fatigue resistance of the trained muscles (Kraemer et al., 1996; Mackey et al., 2018). Since the training backgrounds of PA and SA differed significantly, they could also have differences in the distribution of the muscle fiber types. However, this cannot be confirmed, because no muscle biopsies were taken, but greater maximal force levels in SA could suggest that they had greater muscle mass and perhaps a greater amount of IIA and I muscle fibers, which could lead to better fatigue resistance during PL. Interestingly, there were no significant differences between PA and NA during present PL, which does not fully support the effect of the specific training background. Since NA had very limited experience with resistance exercise, it is possible, that even the total volume of the present PL was quite heavy for them, and led to significant decrements in the neuromuscular performance, even though they do not have earlier training experience with PL types of resistance exercises.

The reductions in MIVC_LP_, AP_SQ_, and CMJ occurred concurrently with significant decreases in both isometric and dynamic sEMG in all groups and VA from Pre to Post 15 in the combined athlete group suggesting that the central factors might have contributed to some extent to the neuromuscular fatigue during PL and MSL. In accordance with the present study, previous studies have observed reductions in sEMG during isometric contraction after PLs (Linnamo et al., 1998; McCaulley et al., 2009) and even greater reductions after MSL (McCaulley et al., 2009). Interestingly, also SA had a significant decrease in both isometric and dynamic sEMG even though their MIVC_LP_ and AP_SQ_ decreased minimally suggesting that they were able to load their neuromuscular system substantially during PL. However, it should be taken into consideration, that especially during the dynamic movements, large individual variability, and some individual differences in training backgrounds within the group and muscle fiber type, could affect the variation of sEMG activity (Nicholson et al., 2014). In addition, the electrodes can move relative to the muscle, and there can be crosstalk from nearby muscles, changes in skin-electrode contact (Farina et al., 2004), or the coactivation of antagonist muscles (Duchateau and Baudry, 2014). The activity of antagonist's muscles was not measured in the present study, and thus, their possible effect on sEMG activity remained uncertain. Interestingly, there were also significant reductions in sEMG_SQ_ in all groups from Mid to Post, even though AP_SQ_ was maintained. This observation could implicate that during fatiguing loading, synergist muscles (e.g., gluteus, vastus intermedius, and rectus femoris muscles) could have taken a greater role in power production (Nicholson et al., 2014; Ortega-Auriol et al., 2018).

There have been some inconsistencies between studies in VA responses during PL and MSL loadings. For instance, Howatson et al. (2016) did not observe a decrease in the central activation ratio after PL in athletes, even though a decline in the maximal voluntary contraction was observed. Whereas, after MSLs, there have been both reductions in voluntary activation (Peltonen et al., 2013; Thomas et al., 2018) and no changes in the central activation ratio (Brandon et al., 2015; Howatson et al., 2016). It should be taken into consideration that VA measured during the unilateral isometric contractions and single-joint movements do not necessarily illustrate the total magnitude of the central fatigue caused by dynamic and multi-joint exercise (de Haan et al., 2009). Furthermore, during the back-squat exercise, several muscles take part in the movement, and the assessment of VA only from the quadriceps femoris muscle may not necessarily demonstrate the total “true” fatigue. In addition, the different stimulation procedures (singlet vs. doublet stimulus), different stimulation locations (on muscle belly vs. directly to the femoral nerve), and different equations used to calculate VA probably partially explain these conflicting results. Furthermore, in the present study, MIVC_LP_ was less affected by PL, and because the stimulus is delivered during maximal force plateau, it is possible that the ITT method may not be the valid measure of “true” VA (de Haan et al., 2009). In addition, it was interesting that reductions in VA were only observed from Pre to Post 15 after both PL and MSL because typically central fatigue recovers very quickly. After PL, also CMJ and AP_SQ_ declined slightly from Post to Post 15, especially in SA, suggesting that neuromuscular fatigue could be delayed. It is possible that post-activation performance enhancement, especially after the present PL, concealed some of the fatigue in the Post time point (Blazevich and Babault, 2019). In addition, the cool-down of the neuromuscular system after the short rest period again for the “true” maximal neuromuscular performance could have also slightly affected the performance in the Post 15 time point.

There were large acute decrements in neuromuscular variables during HL. Both AP_SQ_ and AF_500_ showed somewhat clearer differences between the groups and greater decreases compared to MIVC_LP_, especially after HL and MSL. The high volume and high intensity of these loadings and the long time under tension probably led to mechanical stress causing damage to the muscle structures. Especially, type II muscle fibers are susceptible to muscle damage (Brentano and Martins Kruel, 2011), which could partially explain the greater decreases observed in the power and rapid force production variables in the present study. Despite large acute decrements in maximal force and rapid force production only minor decreases in sEMG were observed during HL. In previous studies, both decreases (Ahtiainen et al., 2004) and no changes (McCaulley et al., 2009; Walker et al., 2012) in sEMG activity during isometric actions have been observed after HLs. In the present study, there were very large increases in BL after HL, which could lead to increased presynaptic inhibition *via* group III and IV afferents observed as reduced sEMG amplitude (Walker et al., 2012). Furthermore, variations in synergist muscle activity (Ortega-Auriol et al., 2018) and decreases in maximal force levels (Dimitrova and Dimitrov, 2003) during the fatiguing loading could explain the decreases observed in sEMG activity during the present study. On the other hand, during fatigue, the increase in firing frequency and/or muscle unit synchronization could explain no changes or even some increases observed in EMG activity (Dimitrova and Dimitrov, 2003).

The present loadings led to significant acute increases in serum TES, COR, and GH in SA and GH in PA during PL and acute increases in GH and COR in all groups and TES in SA and NA during MSL. These results were consistent with previous studies, which have pointed out that strength-trained athletes are capable to produce greater elevations, for instance, in TES compared to non-athletes due to the resistance training background (Ahtiainen et al., 2004; Tremblay et al., 2004). It could be, that the present athlete groups were able to promote the greater endocrine response due to their greater muscle mass and maximal force and, therefore, also larger volume load. Previous studies have observed rather minimal responses in serum COR after MSLs (Häkkinen and Pakarinen, 1993; Crewther et al., 2008). The increase in COR seems to be related to greater metabolic stress (Kraemer and Ratamess, 2005), and it could be that the relatively high volume and intensity (7 × 3RM) used in MSL in the present study was a sufficient stimulus to cause metabolic stress and could explain the significant increase in serum COR.

The greatest acute hormonal elevations were observed during HL, which is in agreement with several studies (Häkkinen and Pakarinen, 1993; Kraemer and Ratamess, 2005; Bartolomei et al., 2017; Martorelli et al., 2021). During HL serum GH increased significantly only in the athlete groups and significant differences in the GH responses between SA and NA were found from Pre to Mid and Pre to Post. The previous study (Ahtiainen et al., 2004) did not observe statistically significant differences between strength athletes and non-athletes in acute elevations of serum GH during the forced repetition HL. However, the greater overall volume and shorter rest periods during the present HL could explain the greater differences in acute increase of GH. Previous studies have linked the increase in GH with the increase in BL (Häkkinen and Pakarinen, 1993; Walker et al., 2011). This was noted also in the present study in the total group of subjects, since the relative increases in GH during HL correlated significantly with the relative increases in BL, and also in the separate group of SA.

### Recovery After the Loadings

It was hypothesized, that the athlete groups would recover faster compared to NA. Nevertheless, no significant between-group differences were observed in any of the measured variables during the recoveries after three resistance exercise loadings. In some variables, the recovery of NA appeared slightly slower but these differences between the groups were minimal and statistically non-significant. The higher maximal force levels in athletes lead to greater absolute loads and overall volume load during all loadings causing possibly greater mechanical stress in working muscles. The athletic populations have also a greater proportion of type II muscle fibers (Izquierdo et al., 2011; Methenitis et al., 2016), which are especially sensitive to mechanical stress and muscle damage (Brentano and Martins Kruel, 2011). In addition, the athletes are able to perform resistance exercises more intensively, and this way achieve greater acute fatigue during the exercises (Ahtiainen and Häkkinen, 2009; Izquierdo et al., 2011). Therefore, it might be that the recovery of the athletes required more time, which could explain minimal differences observed between the athlete groups and NA during the recoveries from the present loading protocols.

After HL, the recovery of MIVC_LP_ from Post to Post 15 appeared somewhat faster in the athlete groups compared to NA, and this could be due to a faster clearance of lactate/hydrogen ions and other metabolites in athletes, which could hinder the ability of muscles to produce force (Walker et al., 2012). There were no significant recoveries in AP_SQ_ or CMJ from Post to Post 15 after PL or MSL, while after HL a significant recovery was observed in all three groups. This difference between the loadings is probably due to different types of fatigue (central vs. peripheral). Because HL caused mainly peripheral fatigue, the recovery could be faster, because the metabolites (e.g., hydrogen ions) had enough time to dissolve, and the phosphocreatine and ATP storages to restore during 15 min of rest (Walker et al., 2012).

After HL, MIVC_LP_ in NA was still reduced after 24 h of rest, whereas both athlete groups had recovered at this point. In addition, the AP_SQ_ of NA remained declined even after 48 h. These findings could support the hypothesis that athlete groups were able to recover slightly faster after HL. CMJ in SA was still reduced after 24 h after both MSL and HL. The slower recovery after MSL, and especially after HL could be related to high-intensity (3-RM and 10-RM) and a longer time under tension leading to considerable muscle damage (Linnamo et al., 1998; Brentano and Martins Kruel, 2011). Thus, due to a possible greater degree of muscle damage, the ability to produce force and power rapidly remained lowered even for 48 h after the loadings.

Serum TES and COR were recovered to baseline after 24 h in all three groups after all three loadings and no differences between the groups were found. Even after HL, serum hormone levels were recovered to the baseline after 24 h, which indicates that the volume of the present HL (5x10-RM) was reasonable. For instance, the study of Häkkinen and Pakarinen (1993) pointed out, that when the volume of HL is extremely high (10x10-RM), serum resting TES levels remained lowered over 2–3 days. Thus, when the volume of the training session is reasonable, the present study demonstrated that the athletes could perform the next similar resistance exercise loading already after 48 h of rest.

It needs to be pointed out that this study did have some limitations. The main limitation was a small sample size in the present subject groups, which limits the generalizability of these results. In addition, the athletes were “only” national-level athletes and even though their training backgrounds did differ specifically, the differences between international level athlete groups would likely have been even greater. Furthermore, quite a large amount of the test procedures were conducted during each loading, which could produce additional neuromuscular fatigue and possibly affect the present results. Alternatively, the test protocol lasted approximately 5 min, and even though all procedures were performed with very short rest periods, it could be that neuromuscular fatigue caused by the loading sets did diminish to some extent during this period. The present results may have also been influenced to some extent by the use of the measurement of VA in the unilateral isometric condition which might not have been the optimal method to evaluate neural fatigue caused by the present loadings, especially during PL. In addition, the loading protocols were performed in a specific order due to challenges in the scheduling of various loadings and recovery measurements, which could have slightly affected the results of the present study. However, a period of one week of recovery was always assured between the loading protocols to minimize the impact of the previous loading protocol.

The present findings can be utilized to optimize and individualize the programming and periodization of resistance exercises in populations with differing training backgrounds (Jeffries et al., 2022). Our results demonstrated, that a greater number of sets (7 sets vs. 4 sets) during PL led to greater neuromuscular fatigue measured by sEMG of the loaded muscles. Thus, when the aim is to induce neural adaptations, it may be beneficial to properly periodize a greater number of sets in PLs. However, it should be kept in mind, that greater total fatigue does not necessarily translate to the greater adaptations in the long-term training. The neuromuscular performance and hormonal levels were recovered after 24 h after PL in all groups, suggesting that the next training sessions can be organized at this point without any residual fatigue. During MSL, about 4–5 sets might be a sufficient stimulus for the neuromuscular system among participants with the present training backgrounds, and when the number of sets is increased also the metabolic demand increases, and this might extend the recovery period. In general, recovery from MSL occurred after 24 h, but rapid force production may remain slightly declined at this point, especially in SA, and this should be considered while periodizing training programs. The present HL led to substantial decreases in neuromuscular performance, especially in the rapid force production, and elevations in serum hormone concentrations. Full recovery after HL required up to 48 h. The overall volume of one specific resistance exercise loading affects the time course of recovery, and it should be determined carefully depending both on the goal, and the training period in question.

In conclusion, the results of this study indicated that the present three different resistance loading protocols of PL, MSL, and HL led to acute decrements in neuromuscular performance and selected acute responses of serum hormone concentrations. The significant differences observed between our three groups during the PL and HL protocols suggest that the training background may cause a greater magnitude of acute responses both in the neuromuscular and endocrine systems during the present loadings. However, the training background seemed to have a more limited effect on the 2-day recovery period after the present three loading protocols.

## Data Availability Statement

The raw data supporting the conclusions of this article will be made available by the authors, without undue reservation.

## Ethics Statement

The studies involving human participants were reviewed and approved by The Human Sciences Ethics Committee of the University of Jyväskylä. The patients/participants provided their written informed consent to participate in this study.

## Author Contributions

JK and ST contributed to acquisition and analysis of data. JK, KH, HP, and SW interpreted the data. All authors concepted and designed the study, drafted, edited, and reviewed the manuscript. All authors have read and approved the final version of the manuscript.

## Conflict of Interest

The authors declare that the research was conducted in the absence of any commercial or financial relationships that could be construed as a potential conflict of interest.

## Publisher's Note

All claims expressed in this article are solely those of the authors and do not necessarily represent those of their affiliated organizations, or those of the publisher, the editors and the reviewers. Any product that may be evaluated in this article, or claim that may be made by its manufacturer, is not guaranteed or endorsed by the publisher.
